# Mortality Statistics

**Published:** 1874-09

**Authors:** 


### *Chicago Mortality Report for July, 1874.*

MORTALITY IN MONTH OF JULY, 1874.

| | | | |
|---|---|---|---|
| Accident, by burns | 3 | Cholera infantum | 594 |
| " by being crushed | 1 | " morbus | 11 |
| " by drowning | 16 | Consumption | 40 |
| " by explosion | 1 | Convulsions | 180 |
| " by fall | 3 | Croup | 3 |
| " in mill | 1 | Colic, lead | 1 |
| " fractured skull | 1 | Cyanosis | 1 |
| " thrown from wagon | 1 | Debility, general | 9 |
| " run over by wagon | 1 | Delirium tremens | 3 |
| " run over by bus | 1 | Diabetes | 1 |
| " by scalding | 1 | Diarrhœa | 67 |
| " by suffocation | 1 | " chronic | 4 |
| " by shooting | 3 | Diphtheria | 4 |
| " by railroad | 2 | Dropsy, general | 6 |
| Abscess, hepatic | 1 | " ovarian | 1 |
| Addison's disease | 1 | Dysentery | 10 |
| Anæmia | 1 | " chronic | 2 |
| Aneurism of aorta | 1 | Dyspepsia | 2 |
| Angina pectoris | 1 | Endo carditis | 1 |
| Apoplexy | 7 | Enteritis | 25 |
| Ascites | 1 | Entero colitis | 33 |
| Atelectasis pulmonum | 1 | Endo metritis | 1 |
| Bladder, hemorrhage of | 1 | Erysipelas | 4 |
| Bowels, hemorrhage of | 1 | Fever, congestive | 2 |
| " ulceration of | 1 | " intermittent | 3 |
| " strangulation of | 1 | " puerperal | 7 |
| Brain, congestion of | 12 | " scarlet | 12 |
| " inflammation of | 12 | " typhoid | 10 |
| " softening of | 1 | Gangrene | 1 |
| Bronchitis | 4 | Gastritis | 5 |
| " capillary | 2 | Gastro enteritis | 16 |
| Caries | 1 | Hæmetemesis | 1 |
| Cancer | 3 | Hemorrhage | 2 |
| " of breast | 4 | Heart, disease of | 5 |
| " of neck | 1 | " dropsy of | 2 |
| " of liver | 1 | " fatty degeneration of | 1 |
| " of stomach | 5 | " valvular disease of | 3 |
| " of uterus | 2 | Hepatitis | 2 |
| Catalepsy | 1 | Hypertrophy | 2 |

| | | | |
|---|---|---|---|
| Hydrocephalus | 22 | Pneumonia | 17 |
| Inanition | 34 | Pleurisy | 2 |
| Intemperance | 4 | Pyæmia | 2 |
| Influenza | 1 | Rheumatism | 2 |
| Intussusception | 1 | Suicide, by poison | 1 |
| Icterus | 1 | " by shooting | 1 |
| Jaundice | 1 | Scrofula | 3 |
| Kidneys, disease of | 2 | Scurvy | 1 |
| " fatty degeneration of | 1 | Small-Pox | 7 |
| Liver, cirrhosis of | 1 | Spine, disease of | 1 |
| " fatty degeneration of | 2 | Stomach, hemorrhage of | 1 |
| Lungs, congestion of | 4 | Stomatitis | 1 |
| Malformation | 1 | Sun stroke | 7 |
| Manslaughter | 2 | Tabes mesenterica | 23 |
| Menstrual suffusion | 1 | Teething | 4 |
| Metro peritonitis | 1 | " and complications | 12 |
| Measles | 4 | Tetanus | 2 |
| Meningitis | 19 | Trismus | 2 |
| " cerebro-spinal | 8 | Tumor | 2 |
| " tubercular | 4 | " of abdomen | 2 |
| Myelitis | 1 | Ulcers, gangrenous | 1 |
| Necrosis | 1 | " and stricture of rectum | 1 |
| Old age | 11 | Uræmia | 1 |
| Paralysis | 1 | Uræmic poisoning | 1 |
| Perimetritis | 3 | Uterus, inflammation of | 2 |
| Peritonitis | 3 | Vitality deficient | 3 |
| " puerperal | 1 | Whooping cough | 17 |
| Perityphlitis | 1 | Total | 1454 |

| | |
|---|---|
| Premature births | 12 |
| Still-births | 57 |
| Total | 69 |

COMPARISON.

| | | |
|---|---|---|
| Deaths in month of July, 1874 | | 1454 |
| " " June, 1874 | | 542 |
| Increase | 912 | |
| Deaths in month of July, 1873 | | 1504 |
| Decrease | 50 | |

AGES.

| | | | |
|---|---|---|---|
| Under one year | 894 | Forty years to fifty | 51 |
| One year to two | 222 | Fifty " " sixty | 33 |
| Two years to three | 39 | Sixty " " seventy | 32 |
| Three " " four | 10 | Seventy " " eighty | 11 |
| Four " " five | 7 | Eighty " " ninety | 4 |
| Five " " ten | 19 | Ninety " " one hundred | -- |
| Ten " " twenty | 43 | | |
| Twenty " " thirty | 43 | Total | 1454 |
| Thirty " " forty | 46 | | |

| | | | |
|---|---|---|---|
| Colored | 5 | Males | 765 |
| White | 1449 | Females | 689 |
| Total | 1454 | Total | 1454 |

NATIONALITIES.

| | | | |
|---|---|---|---|
| Belgium | 1 | New Foundland | 1 |
| Bohemia | 9 | Norway | 11 |
| Canada | 7 | Poland | 2 |
| Native—Chicago | 224 | Prince Edward Island | 1 |
| Foreign, " | 896 | Shetland Isles | 1 |
| United States, other parts | 123 | Scotland | 4 |
| Denmark | 2 | Sweden | 8 |
| England | 15 | Switzerland | 2 |
| Germany | 80 | Unknown | 5 |
| Ireland | 61 | | |
| Italy | 1 | Total | 1454 |

MORTALITY BY WARDS, JULY, 1874.

| Wards. | No. Deaths. | Pop. in 1872. | Percentage. |
|---|---|---|---|
| 1 | 1 | 398 | one death in ---- |
| 2 | 6 | 2,174 | " " ---- |
| 3 | 27 | 19,157 | " " 709 |
| 4 | 16 | 16,832 | " " 1,652 |
| 5 | 32 | 18,564 | " " 580 |
| 6 | 132 | 31,371 | " " 207 |
| 7 | 185 | 27,644 | " " 149 |
| 8 | 138 | 30,253 | " " 219 |
| 9 | 110 | 30,032 | " " 273 |
| 10 | 27 | 16,624 | " " 614 |
| 11 | 29 | 18,340 | " " 632 |
| 12 | 45 | 20,876 | " " 464 |
| 13 | 39 | 14,636 | " " 375 |
| 14 | 41 | 15,892 | " " 388 |
| 15 | 234 | 40,047 | " " 171 |
| 16 | 94 | 19,099 | " " 203 |
| 17 | 68 | 17,513 | " " 258 |
| 18 | 82 | 19,977 | " " 243 |
| 19 | 15 | 2,944 | " " ---- |
| 20 | 22 | 5,023 | " " ---- |
| | 1,343 | Ratio of deaths to population in 1872, one death in 246. | |

| | | | |
|---|---|---|---|
| No. of deaths in Wards | 1,343 | Manslaughter | 2 |
| Accidents | 36 | Protestant Orphan Asylum | 2 |
| Bridewell | 2 | Small Pox Hospital | 2 |
| County Hospital | 7 | St. Joseph's " | 6 |
| County Jail | 1 | St. Luke's " | 5 |
| Foundlings' Home | 36 | Suicide | 2 |
| Hospital Alexian Brothers | 5 | | |
| Hospital for Children | 1 | Total | 1,454 |
| Mercy Hospital | 4 | | |

